# Distance-Decay and Taxa-Area Relationships for Bacteria, Archaea and Methanogenic Archaea in a Tropical Lake Sediment

**DOI:** 10.1371/journal.pone.0110128

**Published:** 2014-10-20

**Authors:** Davi Pedroni Barreto, Ralf Conrad, Melanie Klose, Peter Claus, Alex Enrich-Prast

**Affiliations:** 1 Instituto de Microbiologia Prof. Paulo de Góes, Universidade Federal do Rio de Janeiro, Rio de Janeiro, Brazil; 2 Instituto de Biologia, Universidade Federal do Rio de Janeiro, Rio de Janeiro, Brazil; 3 Max-Planck Institute for Terrestrial Microbiology, Marburg, Hessen, Germany; 4 Department of Water and Environmental Studies, Linköping University, Linköping, Sweden; Linneaus University, Sweden

## Abstract

The study of of the distribution of microorganisms through space (and time) allows evaluation of biogeographic patterns, like the species-area index (z). Due to their high dispersal ability, high reproduction rates and low rates of extinction microorganisms tend to be widely distributed, and they are thought to be virtually cosmopolitan and selected primarily by environmental factors. Recent studies have shown that, despite these characteristics, microorganisms may behave like larger organisms and exhibit geographical distribution. In this study, we searched patterns of spatial diversity distribution of bacteria and archaea in a contiguous environment. We collected 26 samples of a lake sediment, distributed in a nested grid, with distances between samples ranging from 0.01 m to 1000 m. The samples were analyzed using T-RFLP (Terminal restriction fragment length polymorphism) targeting *mcrA* (coding for a subunit of methyl-coenzyme M reductase) and the genes of Archaeal and Bacterial 16S rRNA. From the qualitative and quantitative results (relative abundance of operational taxonomic units) we calculated the similarity index for each pair to evaluate the taxa-area and distance decay relationship slopes by linear regression. All results were significant, with *mcrA* genes showing the highest slope, followed by Archaeal and Bacterial 16S rRNA genes. We showed that the microorganisms of a methanogenic community, that is active in a contiguous environment, display spatial distribution and a taxa-area relationship.

## Introduction

The biogeography concept is defined as the study of the distribution and the range of living organisms across space and time. Most studies in this field were traditionally performed targeting macro-organisms such as plants and animals [1]. Since the development of molecular tools, the concepts of biogeography started to be also studied in microorganisms [2]–[5].

A long-held concept in microbial ecology is that microorganisms are ubiquitously distributed and can be found in any habitat with favorable environmental conditions. This concept was introduced by Martinus Willem Beijerinck and concisely summarized by Lourens Gerhard Marinus Baas Becking in the quote, “Everything is everywhere, the environment selects” [6]. This statement is based on some traits of the microorganisms, such as the small size of individuals and the consequent ease of their dispersal across long distances, high rates of reproduction, short generation times, and large population sizes, leading to a small chance of local extinction.

Free-living eukaryotic microorganisms are often described as occurring ubiquitously. When they are not dominant in some specific environment, it is possible to reanimate the cryptic diversity by changing the environmental conditions *in vitro* [3], [7]. A study showed that it is possible to find nearly 80% of all known species of the flagellate genus *Paraphysomonas* in just a small sample of sediment [8], meaning that the global diversity of this genus is well represented by its local diversity. This observation is mostly explained by the high dispersal rate of the flagellates (due to their low size), their extremely short generation times (leading to a low rate of extinction) and also their capacity to generate resistant forms when the environmental conditions are unfavorable [8]. The authors suggested that if eukaryotic microorganisms were ubiquitous, then prokaryotic microorganisms should be ubiquitous as well, since they have an even smaller size and larger populations. Indeed, some studies on prokaryotes suggested global distribution, for example, psychrophilic polar bacteria were found at both the South and the North poles [9].

Recent studies, however, showed that the distribution of microorganisms is not random, and that biogeography patterns of distribution are established [10], [11]. For example, the genetic distances between populations of microorganisms were shown to increase with geographic distance, which might represent a speciation process driven by the geographic isolation of the microorganisms [12]. Other studies were able to identify endemic microorganisms, and true geographic isolation in extreme environments like hot springs, pristine soils, salt lakes, and hot and cold deserts around the world, all being strong evidence for non-cosmopolitan distribution [10], [13]–[17]. The main difficulty is to evaluate the factors determining the geographical distribution, whether historic evolutionary events (geographic barriers for example) or contemporary ecological environmental factors. [5].

The taxa-area relationship is one of the most consistent general patterns in ecology and well described for macro-organisms [1]. It is represented by the equation **S = c A^z^**, where **S** is the number of species, **A** is the area sampled, **c** is a constant that is empirically derived from the taxon and the specific location studied, and the exponent **z**, the power law index (i.e. **z**-value), represents the rate of the increasing number of species along the increasing sampling area (graphical slope). When significant, the z-value may be strong evidence for biographical distribution of the species.

Values for the z exponent have already been described for microorganisms. Interestingly, z-values for microorganisms were often smaller than for macro-organisms. This result may be attributed: to the larger capacity of dispersion; to the lack of a clear “species” resolution; and to the use of molecular fingerprint or sequencing techniques [18]–[20]. Molecular fingerprint methods, such as T-RFLP (Terminal restriction fragment length polymorphism) and DGGE (Denaturing gradient gel electrophoresis), proved to be an important tool for accessing the diversity of microorganisms in different environments with relatively low costs and little time consumed [21]. However, fingerprinting methods usually are limited as they detect the most common species and thus underestimate the total diversity in a sample [22]. That is mostly because fingerprinting techniques lump different closely related “species” into a single taxonomic unit (often called Operational Taxonomical Unity – OTU), and usually ignore rare species. Nevertheless, fingerprinting techniques are still extremely valuable for rapidly comparing the microbial community composition in different environments [2].

Another parameter of species distribution through space is the distance-decay relationship, which consists of the decay of similarity between different communities as a function of the distance separating them [23], and can also be seen as evidence of a biogeographical pattern. The main difference between the distance-decay approach and the species-area relationship is based on the consideration of the relative abundance of the species in addition of their presence or absence. Bell et al. showed that bacteria in water-filled tree holes, found at the same place, displayed a significant distance-decay relationship [24].

Little is known about the geographical distribution of methanogenic archaea. So far, hot desert soil methanogenic Archaea were shown to be widely spread between different parts of the globe, and this could be found mostly by reactivation of the cryptic methanogenic process *in vitro* [25]. To our best knowledge, there is no description of distance-decay or species-area relationship for non-extremophilic archaea in a contiguous environment. Given the high ecological stability of methanogenic sediments and soils, with a continuous anaerobic environment and a regular input of organic matter, the microbial communities related to the methanogenesis processes tend to be stable through time [26].

Among the processes commonly underlying the biogeographical patterns of distribution of organisms and communities - selection, drift, dispersion and diversification - selection and dispersion are closely related with geographical distances, given the increase of habitat heterogeneity and dispersion limitations with increasing area [27]. Thus, we hypothesized that if there is a change of diversity patterns among different spots of a contiguous lake sediment, it should have a strong correlation with the geographical distance between them, and this change should also present itself differently depending on which part of the microbial community is considered. For this purpose we were targeting three different genes. The 16S rRNA genes of Bacteria and Archaea that are transcribed to generate the structural RNA of the small-subunit ribosomes are universal and strongly conserved genes and are therefore widely used as taxonomic markers [28]. The *mcrA* is a functional gene coding for the alpha subunit of the methyl-coenzyme M reductase, an enzyme being essential and characteristic for the methanogenesis biochemical pathway in Archaea [29]. Thus, we were targeting different groups of prokaryotes. By that we expected to see if there is a differential influence of geographic distance as a factor driving distribution of these three different groups of microorganisms.

We were able to show a geographical distribution pattern of the composition of Bacteria, Archaea and methanogenic Archaea in a contiguous tropical lake sediment and the presence of a significant **z-value** for all three groups. We hypothesized that intrinsic ecological differences between the communities, general diversity profiles, and technical particularities and limitations could be defining the differences in the distribution of these taxonomical groups or at least how we perceived it.

## Materials and Methods

### Sampling and Study Area

The Pantanal consists of the largest floodplain in South America and is periodically flooded by the Paraguay River and its tributaries. Altitude above sea level varies from 80 to 120 m and the total estimated area is around 138.123 km^2^. The water flow continuously carries organic material, and during the flood period the sediment spreads all over the plain constituting the most important source of carbon and nutrients for the methanogenic archaea [30]. The climate is hot and wet in the summer, and cold and dry in the winter. The maximum temperature often surpasses 40°C. Between the months of May and July, the average temperature drops below 20°C, and the minimum temperature may reach 0°C [31].

The study was conducted in a single lake (19°02.651′S 57°30.254′W) called *Lagoa Negra*. No special permission is needed for sediment sampling in non-protected areas in Brazil, so no specific permissions were required for these locations. This study did not involve endangered or protected species.

**Figure 1:**
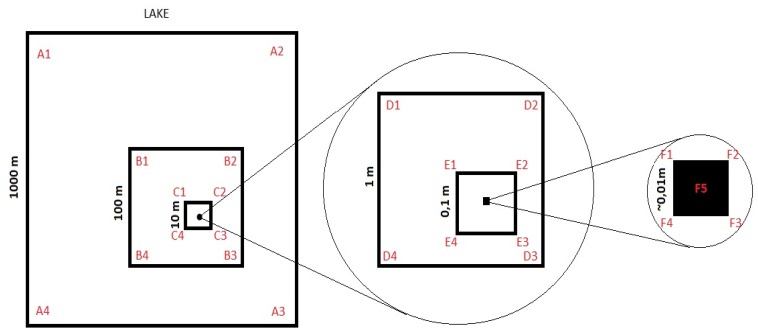
Sampling grid. Designed inside the Lago Negro area, A1-F5 represents the sampling points. (Adapted from (Horner-Devine 2004)).

The lake is located at the west margin of the Paraguay River and a few kilometers east from the cities of Corumbá and Ladário, close to the Bolivia-Brazil border. *Lagoa Negra* is a perennial shallow freshwater lake, with water depth varying between 2 m and 3 m approximately, getting lower or higher following the flood regime. The total area of the lake is 10.8 km^2^ (maximum length of 4.1 km and maximum width of 3.5 km, but it can also vary following the Paraguay River flood regime), water pH ranging from 6.9 to 8.3 and conductivity ranging from 140 to 240 µS cm^−1^ at 25°C. The top layer of sediment is mostly composed of a thin clayey substrate with approximately 2% of sand (>63 µm) and total carbon and organic matter concentrations of 1.8% and 2% respectively [32]. Twenty one sediment cores (7 cm diameter) were collected in the lake in July 2011. The cores were distributed on a nested square grid with distance between points varying from 0.01 m to approximately 1,400 m (Figure 1). The 10 cm top layer of the sediment was homogenized and then 1 ml was sampled and frozen for molecular biology analyses. The central core E was sub sampled within distances of 10 cm and 1 cm between points inside the core area (also 10-cm deep with polyethylene straws). The sediment samples were immediately frozen in liquid nitrogen and then air-shipped to Germany for later molecular biology analyses at the Max-Planck Institute for Terrestrial Microbiology.

### T-RFLP

The frozen sediment samples were thawed, and the total DNA was extracted using the FastDNA SPIN Kit for Soil (MP Biomedicals) following the manufacturer's protocol and a three times treatment with 5.5 M guanidine thyocianate as an additional cleaning step during the matrix binding step. The total DNA was quantified using spectophotometry. Preparations were considered to be of good quality when resulting in more than 30 ng/µl of DNA.

Bacterial and Archaeal 16S rRNA gene fragments were PCR-amplified using the primer pairs Eub 9/27f (5′–GAG TTT GAT CMT GGC TCA G–3′) and Eub 907/926r (5′–CCG TCA ATT CMT TTR AGT TT–3′), as described by Lane and Weisburg [33] for Bacteria, and the primer pairs A109f (5′–ACK GCT CAG TAA CAC GT–3′) and A934b (5′–GTG CTC CCC CGC CAA TTC CT–3′), as described by Großkopf et al. [34] for Archaea. For the T-RFLP analyses the forward Bacteria and the backward Archaea primers were labeled with FAM (5-carboxyfluorescein). Each 50-µl volume of PCR reaction contained 1× GoTaq Flexi Green Buffer (Promega); 1.5 mM MgCl_2_ (Promega); 200 µM of dNTP’s Mix (Fermentas); 0.33 µM of each primer described (Sigma); 1 U GoTaq Flexi DNA Polymerase (Promega) and 10 µg of Bovine Serum Albumin BSA (Roche). Diluted total DNA extract was added as template (1 µl). The reaction was started with a initial denaturation step (94°C for 3 min), followed by 24–28 cycles of denaturation (94°C for 45 sec), annealing (52°C for 45 sec) and extension (72°C for 80 sec), and a final extension step (72°C for 5 min).

For amplification of *mcrA* the primer pair MCRf (5′–TAY GAY CAR ATH TGG YT–3′) and MCRb (5′–ACR TTC ATN GCR TAR TT–3′) was used as described by Springer et al. [29], the forward primer being labeled with FAM for T-RFLP analyses. Each 50-µl of PCR reaction contained 1× MasterAmp PCR PreMix B (Biozym); 0.33 µM of each primer described; 1 U GoTaq Flexi DNA Polymerase (Promega); 10 µg BSA (Roche) and 1 µl of DNA template. The reaction started with an initial denaturation step (94°C for 3 min), followed by 32 cycles of denaturation (94°C for 45 sec), annealing (50°C for 45 sec) and extension (72°C for 90 sec), finished by a final extension step (72°C for 5 min).

The quality of the PCR products was controlled using agarose gel (1.5%) electrophoresis. The DNA was then purified using the GenElute PCR Clean-Up Kit (Sigma) following the manufacturer's instructions, and stored at −20°C.

For T-RFLP, the PCR product was digested using the following restriction enzymes: MspI incubated overnight at 37°C for Bacterial 16S rRNA genes, TaqI incubated for 3 h at 65°C for Archaeal 16S rRNA genes, and Sau96 incubated for 3 h at 37°C for *mcrA*. After the incubation, the digested products were once again cleaned with the SigmaSpin Sequencing Reaction Clean-Up, Post-Reaction Purification Columns (Sigma). The samples were denatured at 94°C for 2 min and loaded into an ABI 3100 automated gene sequencer (Applied Biosystems) for separation of the TRFs. T-RFLP data were retrieved by comparison with an internal standard using GeneScan 3.71 software (Applied Biosystems).

### Taxa-area and distance-decay relationships

The T-RFLP profiles were analyzed and standardized as described in Dunbar et. al [35] resulting in T-RFs of 60 to 855 base pairs (bp) size, each representing more than 1% of the total fluorescence of that sample.

We used the 25 resulting profiles for pair wise calculation of the Bray-Curtis dissimilarity indices [36] and the Sørensen similarity indices resulting in 300 different pairs for each targeted gene. A simple linear regression of the log-transformed data of the Bray-Curtis indices plotted against the distances between the sediment samples from which the pairs originated was used to estimate the slope of the distance-decay relationship [23], in this work we are using the resulting regression coefficients **(Y)** as a parameter for discussion.

The same log-transformed linear regression was used to calculate the slope of the Sørensen indices plotted against the distance between points. The resultant similarity slope was used to calculate the **z-value** of the taxa-area relationship with the formula **log(S_S_) = constant –2** ***z*** **log(D)**, where S_S_ is the pair-wise similarity between communities and D is the distance between two samples used in the distance-decay approach, where the **z-value** is determined by **–(regression coefficient)/2** as described by Harte et al. [37].

By using both, the taxa-area relationship based on the Sørensen index and the distance-decay based on the Bray-Curtis dissimilarity index we addressed the subject not only by “presence/absence” of a taxon (Sørensen index), but also the relative abundance of a taxon (Bray-Curtis index) provided by the T-RFLP.

In order to avoid randomization patterns that could influence the results, the same calculations were performed utilizing distances smaller than 200 m and 20 m and they showed similar slopes as the complete data set (data not shown).

## Results

**Figure 2:**
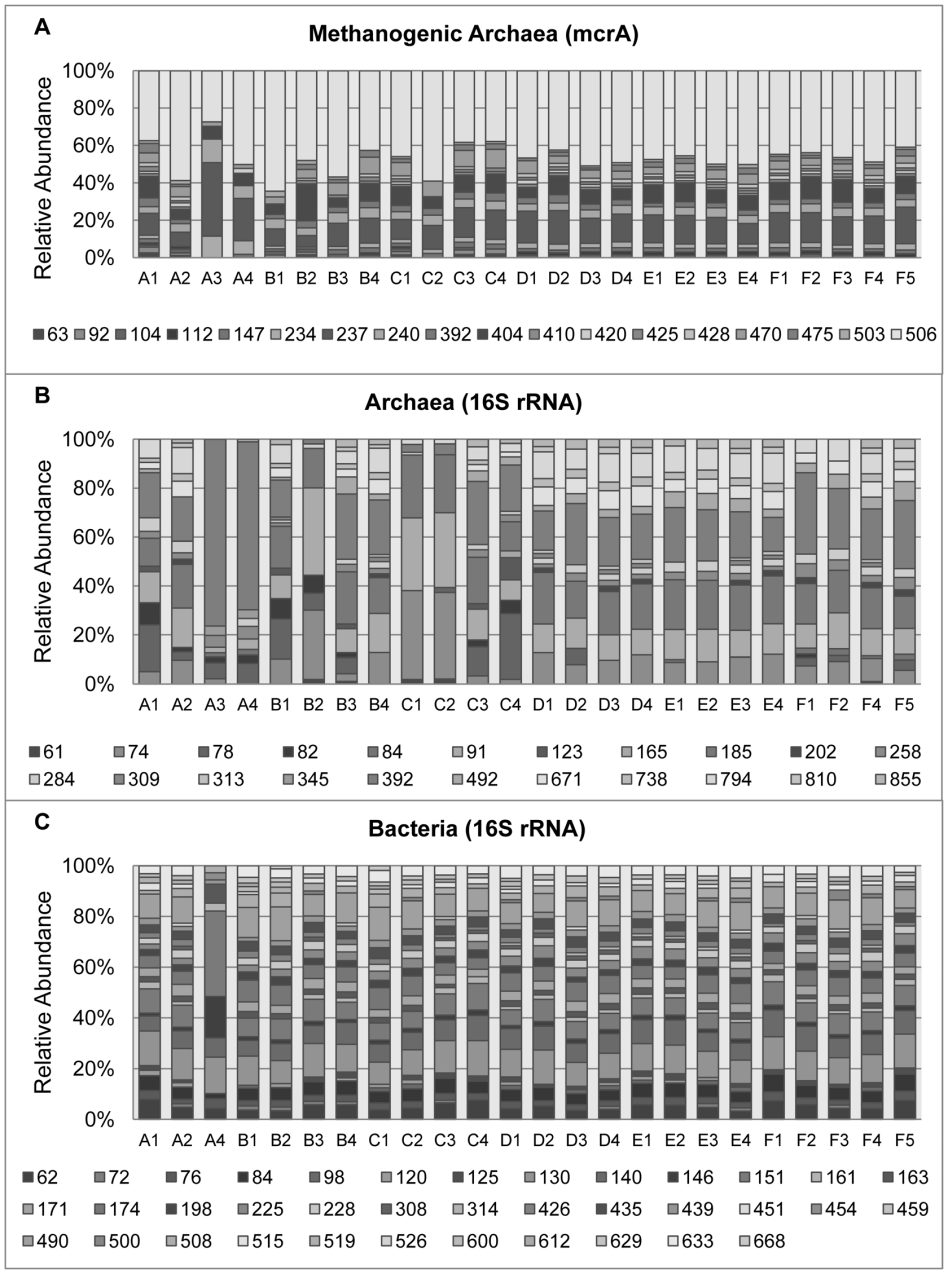
T-RFLP profiles of the three different genes studied in sediment inside the Lago Negro area. A1-F5 represent the sampling points (see Figure 1 for more details), the bar size represents the relative abundance of each OTU, which are defined by their size in base pair (bp). (A) *mcrA*, (B) Archaeal 16S rRNA gene, and (C) Bacterial 16S rRNA gene.

The analysis of the *mcrA* T-RFLP profile resulted in a total of 18 different OTUs of methanogens throughout the lake area (Figure 2A) of which 5 OTUs were found at all sampling points (237 bp, 240 bp, 404 bp, 470 bp and 506 bp) and one OTU (63 bp), which was the least frequently retrieved OTU, was found at only 6 sampling points. The OTU with 506 bp showed the highest relative abundance in all the samples. The Archaeal 16S rRNA T-RFLP profile showed 22 different OTUs (Figure 2B) with none of them showing dominance over the others, only 2 OTUs were found at all sampling points (91 bp and 392 bp) and the OTUs with 165 bp and 345 bp were found at only 1 and 2 sampling points, respectively. The Bacterial 16S rRNA gene T-RFLP profiles showed the largest number of OTUs among the different genes targeted with a total of 37 different OTUs found at all the sampling points across the lake area, showing a high diversity of dominant groups (Figure 2C). Eight OTUs were recovered from all the sampling points (62 bp, 76 bp, 84 bp, 130 bp, 140 bp, 146 bp, 151 bp and 439 bp); in contrast, 3 other OTUs (459 bp, 526 bp and 612 bp) were recovered from only 3 sampling points. In a literature based affiliation it was possible to relate some of the observed OTUs with previously described ones, keeping in mind that such affiliation can only be tentative (Table S1).

Linear regressions were performed to evaluate the slope coefficient of the correlations between the geographical distance and the communities’ similarities, and Mantel tests (with 9999 permutations) were performed to test the significance of these correlations.

**Figure 3:**
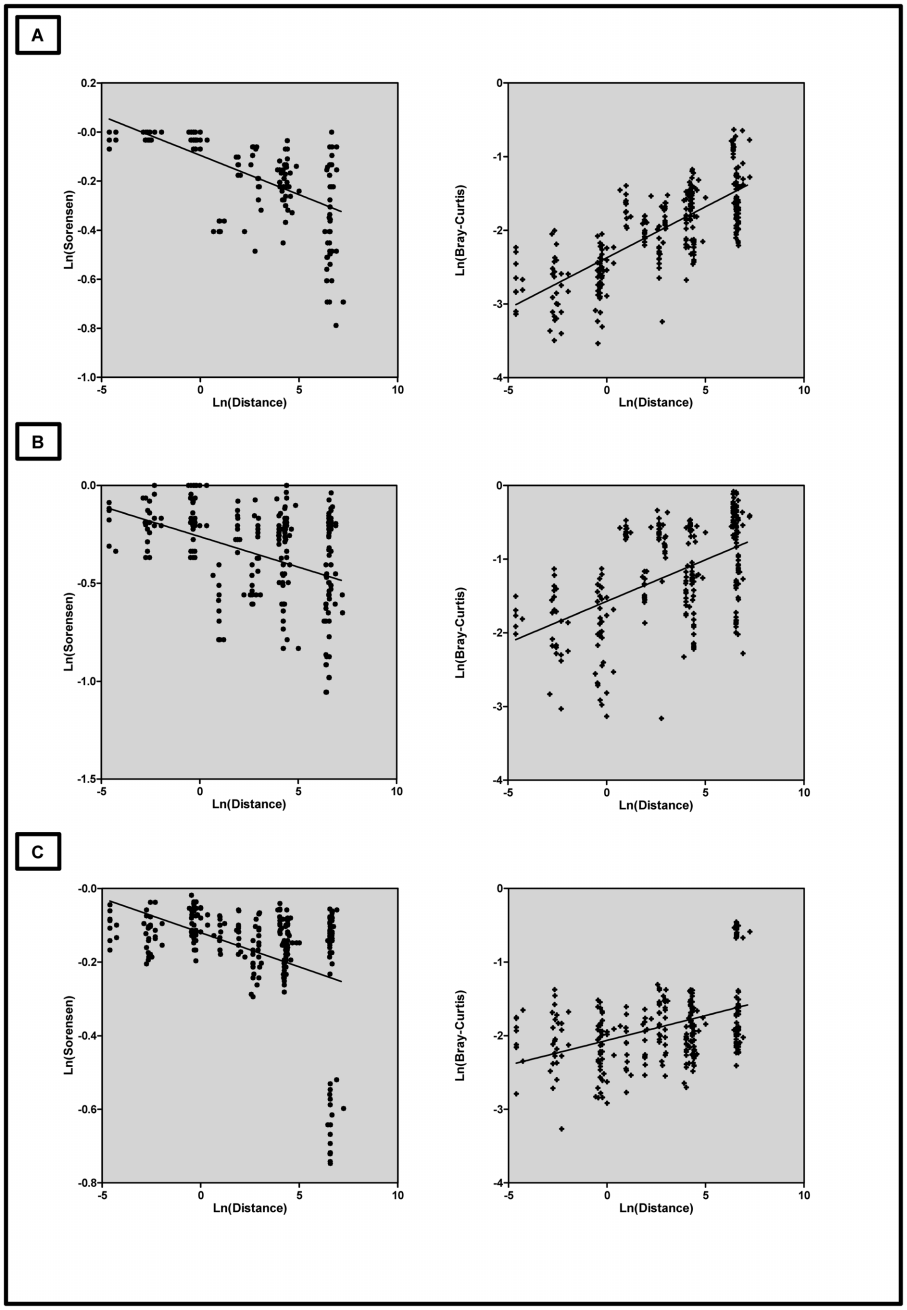
Distance decay and taxa-area relationships. Log transformed Bray-Curtis and Sørensen indices plotted against the distance separating the points, for the three different genes, lines represent a simple linear regression. (A) *mcrA*; (B) Archaeal 16S rRNA gene and (C) Bacterial 16S rRNA gene.

All the three different T-RFLP profiles showed a significant distance-decay relationship based on the Bray-Curtis dissimilarity **(Y)** (Figure 3). The *mcrA* gene showed the largest slope of 0.14 (Mantel test P<0.001, R = 0.69), followed by the archaeal 16S rRNA gene with a slope of 0.11 (Mantel test P = 0.005, R = 0.30), while the slope for the bacterial 16S rRNA gene was only 0.067 (Mantel test P = 0.006, R = 0.34).

The regression coefficient based on the Sørensen similarity indices calculated for all the three genes also showed significant values (Figure 3) of −0.032 (P<0.001, R = −0.60) for *mcrA*, −0.031 (Mantel test P = 0.03, R = −0.45) for archaeal and −0.018 (Mantel test P = 0.006, R = −0.34) for bacterial 16S rRNA genes. However the calculated z-values were small, thus indicating relatively flat taxa-area relationships (Table 1).

**Table 1:** Distance-decay regression coefficients based on the Bray-Curtis dissimilarity index **(Y)**; Regression coefficient based on the Sørensen Similarity index; and the exponent **z** calculated by the distance-decay approach values for each one of the target genes calculated by -(regression coefficient)/2.

*Gene*	*Distance-decay (Y)*	*Sørensen Regr. Coefficient*	*(z)*
***mcrA***	0.14	−0.032	0.016
**Archaea 16S rRNA**	0.11	−0.031	0.0155
**Bacteria 16S rRNA**	0.067	−0.018	0.009

## Discussion

Our study indicates a small but significant biogeographical distribution of the microbial community composition in the lake sediment for all the three different genes targeted, which represent the Bacteria, the Archaea, and the methanogens. The conclusion is based on the distance decay relationship of operational taxonomic units of three different microbial genes. All dominant operational taxa (more than 10% relative abundance) from the three genes analyzed in this study were observed at all the sampling points within the lake. Nevertheless, a significant taxa-area relationship could be observed. These values were small if compared to others described for macro-organisms, which was expected given the high capacity of dispersion of the targeted microorganisms. However, the similarity distance-decay, which takes into consideration also the relative abundance of the groups, shows that they were not homogeneously distributed within the entire lake area. We therefore conclude that the most distant samples were the ones, which were most different from each other.

The **z-values** and the distance decay regression coefficients **(Y)** observed in this study were lower than others previously observed for microorganisms in the literature [17]–[19] [36]–[38], but are the first ones described for Bacteria, Archaea and methanogenic archaea in a single contiguous environment. The existence of significant **z-values** shows that all these microbial groups have a biogeographical distribution. The low values that we found were expected given that we used a fingerprint technique for accessing the microbial diversity of our target environment, and not a high resolution ribotyping technique. At an OTU resolution of 95% sequence similarity of the 16S rRNA gene, Horner-Devine et al. described similar z-values for Bacteria (z = 0.019) and Beta-proteobacteria (z = 0.008) in salt-marshes [18]. At higher resolutions the z presented higher values (0.04 for Bacteria and 0.019 for Beta-proteobacteria at 99% sequences similarity). It is interesting to note that the lower values described by the mentioned study were presented by the lower taxonomical levels reached, while in this study we observed higher values for *mcrA* and the Archaea than for the Bacteria. Other **z-values** found for other groups of microorganisms are displayed in the Table 2.

**Table 2:** Z-values previously described for microorganisms, 99% or 95% represent the taxonomic resolution applied for definition of the OTUs (sequence similarity).

Microbial Community	z-value	Diversity Access	Habitat Org.	Reference
Desert Soil Fungi	0.074	fingerprint (ARISA)	Contiguous	[19]
Salt-Marsh Bacteria (99% OTU)	0.04	sequencing	Contiguous	[18]
Salt-Marsh ß-proteobacteria (99% OTU)	0.019	sequencing	Contiguous	[18]
Salt-marsh Bacteria (95% OTU)	0.019	sequencing	Contiguous	[18]
Salt-Marsh ß-proteobacteria (95% OTU)	0.008*	sequencing	Contiguous	[18]
Water-filled tree holes Bacteria	0.26	fingerprint (DGGE)	Island	[20]
Metal-cutting fluid sump Bacteria	0.26-0.29	fingerprint (DGGE)	Island	[38]
Soil Bacteria	0.03	Fingerprint (T-RFLP)	Noncontiguous	[39]
Tropical Forest Soil Bacteria	0.42 and 0.47	Fingerprint (T-RFLP)	Contiguous	[40]
Freshwater lake sediment Bacteria	0.009	fingerprint (T-RFLP)	Contiguous	this study
Freshwater lake sediment Archaea	0.0155	Fingerprint (T-RFLP)	Contiguous	this study
Freshwater lake sediment methanogenic Archaea	0.016	fingerprint (T-RFLP)	Contiguous	this study

*not significant different from zero.

The differences between **z-values** and distance decay values **(Y)** described for the three different genes, representing three different groups of microorganisms, may be explained by two hypotheses. The first one is that Bacteria (16S rRNA) have a larger capacity for dispersal than the Archaea (16S rRNA) in general and the methanogenic archaea (*mcrA*) in particular. By sampling the first 10 centimeters of the sediment we were able to access different communities living at different depths at the same time. We assume that the methanogens (all Archaea) were preferable located in the deeper sediment layers, since their activity is inhibited by the presence of other electron acceptors like oxygen, nitrate, iron and sulfate, potentially present in the surface layers of the sediment. On the other hand, Bacteria were not restricted to deeper sediment layers [41]–[43]. The Bacteria domain showed weaker species-area and distance-decay relationships than the methanogens (*mcrA*) and the Archaea (16S rRNA). The profile of Archaea and Bacteria OTUs was well distributed throughout the lake area, showing the large diversity of micro-habitats that can be exploited by these groups, and that the ability of dispersion seems not to be compromised at smaller scales.

The second hypothesis is based on the taxonomic sensitivity of the T-RFLP method. OTUs can represent a large variety of different taxonomic groups. The universal primers targeting 16S rRNA genes do not represent the entire set of conceivable species, which are many more than the OTUs derived from T-RFLP. Hence, the total diversity of the community is underestimated by T-RFLP [44]. A study targeting 16S rRNA genes of the bacterioplankton of temperate lakes showed that the sequence similarity within a single OTU varied from 73 to 100%, however, sequence homology of 97% is generally used to define bacterial species [5], [45]. When utilizing a functional gene such as the *mcrA*, the probability of reaching lower taxonomic levels is higher, because translated genes show diminished conservation and can present a higher variability of codons, and thus a higher chance for being differentiated by terminal fragment size methods [46].

This may explain why the **z-values** were higher for T-RFLP of *mcrA* than of 16S rRNA genes, but it does not explain why the Archaea distance decay and **z-values** were higher than those of the Bacterial since both of them targeted 16S rRNA genes. Several authors described that Bacteria diversity tends to be higher than Archaea diversity at different environments [47]–[49], thus the T-RFLP fingerprinting technique could be more efficient in accessing a larger part of the total diversity of Archaea 16S rRNA and consequentially being closer to the actual taxa distribution of the environment, thus showing higher values of **z** and **Y**.

Our work shows some important results that contribute to a better understanding of similar ones developed within different environments. Studies of bacterioplankton distribution in lakes showed similar patterns between different and not connected lakes in distant geographic regions, but with variations in their relative abundance [50]. On the other hand bacterioplankton communities distance dissimilarity inside one single lake were described as being weaker than between different lakes in North America, and mostly influenced by different water regimes and partial geographic isolation [51]. Some studies in saline lakes in China, Mongolia and Argentina showed that Bacterial biogeography in these environments was based on contemporary environmental factors (Na^+^, CO_3_^2−^, and HCO_3_^−^ ion concentrations, pH and temperature) and geographic distance, while Archaeal biogeography was influenced only by environmental factors [17].

Geographical distances have been previously described as an important factor related with microbial spatial distribution corroborating our findings [52]–[54]. Rosseló-Mora et al. used metabolic compounds as a comparison parameter between different populations of the extremophilic bacterium *Salinibacter rubium*. They discovered that the divergence among different phenotypes was related to different geographical locations, and geographical distance between the sites[55]. Environmental and geographical factors also influenced magnetotactic bacteria biogeographical distribution [56].

Our distance-decay and taxa-area relationships can possibly be a result of some degree of dispersal limitation coupled with ecological drift, thus maintaining the taxa-area relationship and distance-decay pattern significant. It is believed that drift plays a important role for the distance-decay patterns found among microorganisms living in some degree of dispersal limitation (i.e. in subsurface habitats)[5]. A high dispersal rate is expected inside this contiguous environment with no significant geographical barriers imposing any kind of mobility limitation to microorganisms, and that could be a factor flattening the curves. But as we already stated, a group preferentially found in deeper layers of sediment (as the methanogenic archaea) could be more easily restricted.

This study did not focus on scanning environmental factors that could be driving the observed biogeographical pattern, so selection as a possible driving factor cannot be excluded, and selection processes are also related with geographical distances, as the diversity of habitats tends to increase with an increasing area [5], [27]. However, given the small scale of the study and the probable absence of a clear environmental gradient within the Lagoa Negra we don’t think it as a major factor. We believe that the correlation that we described between geographical distance and communities’ structures are representative of the biogeographical pattern of distribution of the lake microbial community.

In conclusion, we showed that there was a significant geographic distribution of methanogenic archaea, Archaea and even Bacteria, related with geographic distance in a contiguous environment, i.e. the sediment of a tropical lake.

## Supporting Information

Table S1 **Tentative genetic affiliation of the OTUs.** For the three studied genes there are in the literature some genetic affiliation with different phylogenetic groups, we show some of these in this table. * The affiliation with the phylogenetic groups was based on literature data that used the same primers and restriction enzymes for TRFLP. All the affiliations are only tentative, and can only be interpreted as the probable main groups of the lake sediment community.
(DOCX)
