# Supplementary material for: Distance-Decay and Taxa-Area Relationships for Bacteria, Archaea and Methanogenic Archaea in a Tropical Lake Sediment
Source: PLoS One. 2014 Oct 20;9(10):e110128. doi: 10.1371/journal.pone.0110128 (PMC4203765; doi:10.1371/journal.pone.0110128)
Supplement: Table S1 — Tentative genetic affiliation of the OTUs. For the three studied genes there are in the literature some genetic affiliation with different phylogenetic groups, we show some of these in this table. * The affiliation with the phylogenetic groups was based on literature data that used the same primers and restriction enzymes for TRFLP. All the affiliations are only tentative, and can only be interpreted as the probable main groups of the lake sediment community. (DOCX) [file pone.0110128.s001.docx]

**Table S1 – Tentative genetic affiliation of the OTUs –** For the three studied genes there are in the literature some genetic affiliation with different phylogenetic groups, we show some of these in this table. * The affiliation with the phylogenetic groups was based on literature data that used the same primers and restriction enzymes for TRFLP.

| Target Gene | TRF Size (bp) | Tentative Phylogenetic Afiliation* | Reference |
| --- | --- | --- | --- |
| *mcrA* | 147 | Methanosaetaceae | [1–4] |
|  | 234 | Methanocellales | [1–4] |
|  | 237 | Methanocellales | [1–4] |
|  | 392 | Methanosarcinaceae | [1–5] |
|  | 404 | Mehanobacteriaceae | [1–3,5] |
|  | 420 | Methanosaetaceae | [2,5] |
|  | 470 | Methanobacteriaceae | [1–4] |
| Archaea 16S rRNA | 74 | Rice Cluster V | [1,6] |
|  | 91 | Methanobacteriaceae, Rice Cluster IV, Rice Cluster VI , LDS | Unpublished |
|  | 185 | Methanosarcinaceae, Rice Cluster VI, Crenarchaeota 1.1b. | [1,4,6] |
|  | 392 | Rice Cluster II, Crenarchaeota 1.3, Methanocellales, Methanomicrobiales | [1,4,6] |
|  | 794 | Rice Cluster V; Methanobacteriales | Unpublished |
| Bacteria 16S rRNA | 76 | Methylocaldum/Polyangium | [7] |
|  | 130 | Bacillus/Geobacter | [7] |
|  | 140 | Nocardiodes/ Rubrivivax | [7] |
|  | 490 | Betaproteobacteria | [7] |
|  | 600 | Nitrospira | [7] |

**References**

1. Chin K-J, Lueders T, Friedrich MW, Klose M, Conrad R (2004) Archaeal community structure and pathway of methane formation on rice roots. Microbial ecology 47: 59–67.

2. Lueders T, Chin KJ, Conrad R, Friedrich M (2001) Molecular analyses of methyl-coenzyme M reductase alpha-subunit (mcrA) genes in rice field soil and enrichment cultures reveal the methanogenic phenotype of a novel archaeal lineage. Environmental microbiology 3: 194–204.

3. Conrad R, Klose M, Noll M, Kemnitz D, Bodelier PLE (2008) Soil type links microbial colonization of rice roots to methane emission. Global Change Biology 14: 657–669.

4. Ramakrishnan B (2001) Archaeal community structures in rice soils from different geographical regions before and after initiation of methane production. FEMS microbiology ecology 37: 175–186.

5. Kemnitz D, Chin K-J, Bodelier P, Conrad R (2004) Community analysis of methanogenic archaea within a riparian flooding gradient. Environmental microbiology 6: 449–461.

6. Chin KJ, Lukow T, Conrad R (1999) Effect of temperature on structure and function of the methanogenic archaeal community in an anoxic rice field soil. Applied and environmental microbiology 65: 2341–2349.

7. Noll M, Matthies D (2005) Succession of bacterial community structure and diversity in a paddy soil oxygen gradient. Environmental microbiology 7: 382–395.
